# RAINBIO: a mega-database of tropical African vascular plants distributions

**DOI:** 10.3897/phytokeys.74.9723

**Published:** 2016-11-07

**Authors:** Gilles Dauby, Rainer Zaiss, Anne Blach-Overgaard, Luís Catarino, Theo Damen, Vincent Deblauwe, Steven Dessein, John Dransfield, Vincent Droissart, Maria Cristina Duarte, Henry Engledow, Geoffrey Fadeur, Rui Figueira, Roy E. Gereau, Olivier J. Hardy, David J. Harris, Janneke de Heij, Steven Janssens, Yannick Klomberg, Alexandra C. Ley, Barbara A. Mackinder, Pierre Meerts, Jeike L. van de Poel, Bonaventure Sonké, Marc S. M. Sosef, Tariq Stévart, Piet Stoffelen, Jens-Christian Svenning, Pierre Sepulchre, Xander van der Burgt, Jan J. Wieringa, Thomas L. P. Couvreur

**Affiliations:** 1 Institut de Recherche pour le Développement (IRD), UMR DIADE, 911 Avenue Agropolis, 34394 Montpellier, France; 2 Laboratoire d’évolution Biologique et Ecologie, Faculté des Sciences, Université Libre de Bruxelles, CP160/12, avenue F.D. Roosevelt 50, 1050 Bruxelles, Belgium; 3 CESAB / FRB, Domaine du Petit Arbois, Av. Louis Philibert, Aix-en-Provence, 13100, France; 4 Institut de Recherche pour le Développement (IRD), UMR AMAP, Boulevard de la Lironde TA A-51 / PS 2 34398 Montpellier, France; 5 Section for Ecoinformatics & Biodiversity, Department of Bioscience, Aarhus University, Ny Munkegade 114, DK-8000 Aarhus C, Denmark; 6 Herbarium et Bibliothèque de Botanique Africaine, Université Libre de Bruxelles, Boulevard du Triomphe, B-1050 Bruxelles, Belgium; 7 Botanic Garden Meise, Nieuwelaan 38, 1860 Meise, Belgium; 8 Royal Botanic Garden Edinburgh, 20A Inverleith Row Edinburgh, United Kingdom; 9 Laboratoire de Botanique Systématique et d’Écologie, École Normale Supérieure, Université de Yaoundé I, PO Box 047, Yaoundé, Cameroon; 10 Missouri Botanical Garden, Africa & Madagascar Department, St. Louis, United States of America; 11 Naturalis Biodiversity Center, Darwinweg 2, 2333 CR Leiden, The Netherlands; 12 Wageningen University, Biosystematics Group, Droevendaalsesteeg 1 6708 PB Wageningen, The Netherlands; 13 Institut für Geobotanik und Botanischer Garten, University Halle-Wittenberg, Neuwerk 21, 06108 Halle, Germany; 14 Laboratoire des Sciences du Climat et de l’Environnement, LSCE/IPSL, CEA-CNRS-UVSQ, Université Paris-Saclay, F-91191 Gif-sur-Yvette, France; 15 Laboratoire d'Ecologie végétale et Biogéochimie, Université Libre de Bruxelles, Boulevard du Triomphe, B-1050 Bruxelles, Belgium; 16 Centre for Ecology, Evolution and Environmental Changes (CE3C), Faculty of Sciences, University of Lisbon, Campo Grande, 1749-016, Lisbon, Portugal; 17 CIBIO/InBio, Centro de Investigação em Biodiversidade e Recursos Genéticos, Universidade do Porto. Campus Agrário de Vairão, Vairão, Portugal; 18 CEABN/InBio, Centro de Ecologia Aplicada “Professor Baeta Neves”, Instituto Superior de Agronomia, Universidade de Lisboa, Tapada da Ajuda, 1349-017 Lisboa, Portugal; 19 Royal Botanic Gardens, Kew, Richmond, Surrey TW9 3AE, UK; 20 Department of Ecology, Faculty of Science, Charles University, Vinicna 7, CZ-12843, Prague, Czech Republic; 21 Picturae, De Droogmakerij 12, 1851LX Heiloo, The Netherlands

**Keywords:** Herbarium specimens, tropical forests, georeferencing, taxonomic backbone, habit, digitization, native species, cultivated species, biodiversity assessment

## Abstract

The tropical vegetation of Africa is characterized by high levels of species diversity but is undergoing important shifts in response to ongoing climate change and increasing anthropogenic pressures. Although our knowledge of plant species distribution patterns in the African tropics has been improving over the years, it remains limited. Here we present RAINBIO, a unique comprehensive mega-database of georeferenced records for vascular plants in continental tropical Africa. The geographic focus of the database is the region south of the Sahel and north of Southern Africa, and the majority of data originate from tropical forest regions. RAINBIO is a compilation of 13 datasets either publicly available or personal ones. Numerous in depth data quality checks, automatic and manual via several African flora experts, were undertaken for georeferencing, standardization of taxonomic names and identification and merging of duplicated records. The resulting RAINBIO data allows exploration and extraction of distribution data for 25,356 native tropical African vascular plant species, which represents ca. 89% of all known plant species in the area of interest. Habit information is also provided for 91% of these species.

## Introduction

Improving our understanding of the distribution of biodiversity has been suggested as “one of the most significant objectives for ecologists and biogeographers” (Gaston 2000). Indeed, fundamental understanding of biodiversity patterns and inference of conservation assessments leading to wise and sustainable management of biodiversity at various scales are heavily dependent on our knowledge of species distributions. For tropical regions especially, we have had an incomplete understanding of species distributions which causes a major problem for ecological and conservation research (Bini et al. 2006, Feeley and Silman 2011). There has been a global lack of tropical biodiversity data availability (Collen et al. 2008, Feeley and Silman 2011), although this is increasingly being improved (e.g., Enquist et al. 2009; http://bien.nceas.ucsb.edu/bien/, www.gbif.org, ter Steege et al. 2016). The tropical vegetation of Africa contains high levels of species diversity but is subject to important shifts in response to ongoing climate change and increasing anthropogenic pressures (Blach-Overgaard et al. 2015; Hansen et al. 2013; Lewis et al. 2009; McClean et al. 2005). Even though our knowledge of plant species distribution patterns in the African tropics has been improving over the years (Linder et al. 2012; Stropp et al. 2016), it remains limited (Küper et al. 2006), which calls for initiatives to collate African biodiversity data.

**Figure 1.**
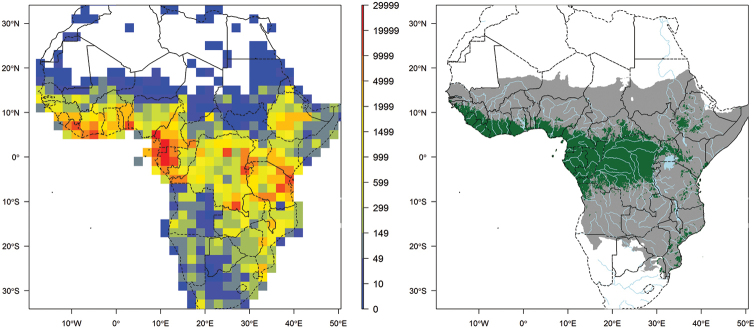
Left map: record density in 2° × 2°cell including all georeferenced records that passed the quality checks. This map includes records that are identified or not to species level. Right map: main extent of RAINBIO geographical coverage from south of Sahel and north of Southern Africa (grey area); extent of tropical rain forest regions adapted from the land cover map published by Mayaux et al. (2004) (green area).

Here, we present RAINBIO, a unique comprehensive database of georeferenced records of vascular plants (Tracheophyta) in sub-Saharan tropical Africa and north of Southern Africa, including Gulf of Guinea islands, Cape Verde and Zanzibar archipelagos (Fig. 1). Until recently, distribution data on tropical African plants were scattered among institutions and individual researchers and not compiled into a single comprehensive database. A recent analysis of African (including Madagascar) vascular plant species occurrences available via the Global Biodiversity Information Facility portal (GBIF, www.gbif.org) resulted in 934,676 herbarium records after data filtering for 57 countries (Stropp et al. 2016). However, over half of these specimens (512,680) belonged to South Africa alone, with Madagascar and Tanzania having the second and third most specimens, respectively (Stropp et al. 2016). This study underlined the lack of high quality data for tropical Africa, especially the forested regions. Several resources, often accessible via the internet, offer access to a large number of occurrences thanks to recent efforts to digitize and georeference herbarium specimens (e.g. TROPICOS, Oever and Gofferje 2012, Heerlien et al. 2014). Additionally, researchers on tropical African botany have created their own “working” datasets for their plant groups or regions of interest (e.g. Blach-Overgaard et al. 2010; Droissart et al. 2012; Wieringa and Sosef 2011). These datasets have the advantage of having updated specimen identifications and generally more accurate georeferencing compared to the larger institutional datasets. RAINBIO is a compilation of thirteen datasets and should be seen as a readily workable dataset because we applied several quality filters, checked the data quality (both georeferencing and taxonomy) and identified and merged duplicate records.

## General description and purpose

The first target of the RAINBIO project (African RAIN forest community dynamics: implications for tropical BIOdiversity conservation and climate change mitigation) funded by CESAB (CEntre de Synthèse et d'Analyse sur la Biodiversité) of the FRB (Fondation pour la Recherche sur la Biodiversité, France), is to compile a state-of-the-art dataset on plant species distribution across tropical Africa. RAINBIO uses large publicly available datasets and smaller “non public”/private databases. The resulting RAINBIO mega database allows the exploration and extraction of distributional data for 25,356 species (29,664 taxa including infraspecific taxa: subspecies and varieties) across continental tropical Africa. It is the first step towards a standardization of plant occurrences in this region and also contributes towards achieving Target 1 of the first Objective of the Global Strategy for Plant Conservation, “*an online flora of all known plants*”, adopted by the Convention on Biological Diversity (Paton et al. 2008).

### Datasets

Two datasets are provided in *csv* format as well as an *R.data* working space (http://rainbio.cesab.org/). For the latter, an R script is provided for exploring and mapping occurrences.

The database made available here represents a subset of available fields (see below). The actual RAINBIO database follows the Darwin Core standard (Wieczorek et al. 2012). Users interested in fields not provided here (see details in http://rainbio.cesab.org/) are invited to contact the first author or last author.

The RAINBIO database is subject to future updates. Users interested in having an updated version of the database are invited to contact the first or the last author.

**Object name**: RAINBIO occurrence database of tropical African vascular plants

**Character encoding**: UTF-8

**Format name**: CSV and R.data

**Format version**: 1.0

**Distribution**: http://rainbio.cesab.org/

**Publication date of data**: September 2016

**Language**: English

**Metadata**: http://vmamapgn-test.mpl.ird.fr/geonetwork/srv/eng/search#|75cf4509-1797-481f-b03c-9dcdce3c773f

**Licenses of use**: This database is made available under license Open Data Commons Attribution: http://www.opendatacommons.org/licenses/by/1.0/

**Elements:**

- RAINBIO unique identifier.
- Unique identifier of the source dataset.
- Taxonomic information (order, family, genus, species and infra-specific taxa).
- Country.
- Geographical coordinates in decimal degrees.

**Object name**: RAINBIO species checklist of tropical African vascular plants

**Character encoding**: UTF-8

**Format name**: CSV and R.data

**Format version**: 1.0

**Distribution**: http://rainbio.cesab.org/

**Publication date of data**: September 2016

**Language**: English

**Metadata**: http://vmamapgn-test.mpl.ird.fr/geonetwork/srv/eng/search#|d20604bc-ce2d-444f-b4b6-e73e55ad3ef2

**Licenses of use**: This database is made available under license Open Data Commons Attribution: https://creativecommons.org/licenses/by-nc/4.0/legalcode

**Elements:**

- Taxonomic information (order, family, genus, species and infra-specific taxa).
- Habit type.

### Collectors and owners of the data

RAINBIO is a compilation of thirteen datasets (more details on these sources at the end of the article) of three kinds: (i) extensive ‘public’ databases of several herbaria institutes (BR, BRLU, K, LISC, MO, and WAG (incl. AMD, L & U as well); acronyms according to Thiers (continuously updated), (ii) personal databases collated by individual researchers (focusing on a given taxonomic group or a given geographic area) and (iii) other sources of plant occurrences such as silica-gel collections or vegetation plot inventories. The WAG dataset includes a series of personal datasets (like ii) compiled for taxonomical revisions of over 35 genera in different families. Occurrences are thus supported by specimens deposited in herbaria (586,920 records), silica-dried specimens (13,510 records) or observations from plot inventories (13,443 records).

### Methods of data collection

The workflow for building the database involved numerous steps of cleaning, standardizing and quality checks described below. These steps were essentially built up in Postgres and PostGis scripts. Several other cleaning and checking steps were run using the R statistical software (R Core Team 2015).

### Georeferencing verification processes

**Figure 2.**
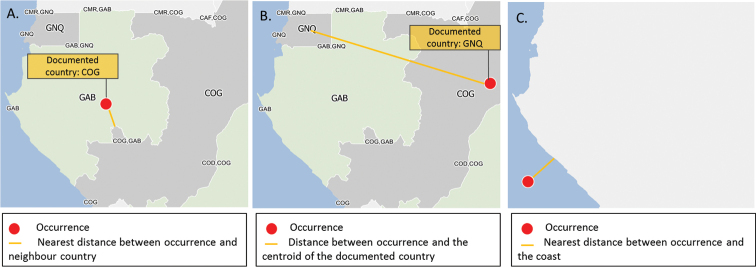
Examples of the georeferencing verification process. **a** The georeferenced record falls within a neighbouring country (here Gabon - GAB) of its documented country (here Republic of the Congo - COG). The nearest distance between the occurrence and the border of the documented country is computed **b** The georeferenced record falls within a non-neighbouring country (here Equatorial Guinea - GNQ) of its documented country (here Republic of the Congo). This record is classified as ‘Error’ and is discarded **c** The georeferenced record lies beyond the coastline. The nearest distance between the occurrence and the coastline of the documented country is computed.

We performed two quality control checks on the geographical coordinates of the records:

First we checked if the documented country of each record corresponds to the country in which the record is georeferenced (Fig. 2).

If false, we checked whether the georeferenced record fell within a country neighbouring the documented country (Fig. 2A). If true, the occurrence was classified as ‘*Neighbour*’ and the nearest distance between the occurrence and the border of the documented country was calculated. Records with a distance of more than 5 km were discarded while records with a distance less than 5 km were retained. The logic behind this is that records could well be from a country neighbouring the one provided by the coordinate, because either the map or the coordinate is not precise enough.

Second, we checked if the occurrence fell within an ocean (Fig. 2C).

If true, the nearest distance between the occurrence and the coastline was calculated. If the distance was greater than 5 km, the record was discarded. If the distance was less than 5 km the record was retained. Again, the logic behind this was that the coordinate or the map may not be precise enough.

If false, the record was discarded.

### Taxonomic backbone and standardization of taxonomic names

To resolve problems such as spelling errors and/or synonymies linked to heterogeneous taxonomic datasets, we first relied on the taxonomic backbone table used by the Naturalis Biodiversity Center herbaria (AMD, L, U & WAG). The structure of this table provides links among taxa names allowing the standardization of species name spelling and synonyms.

We then submitted this taxon list (30,147 names) to the online “Taxonomic Name Resolution Service” (TNRS, Boyle et al. 2013). This tool compares submitted names to names from four different sources (TROPICOS (http://www.tropicos.org/), USDA (http://plants.usda.gov), the Global Compositae Checklist (www.compositae.org/checklist) and The NCBI Handbook (http://www.ncbi.nlm.nih.gov/guide/taxonomy/)). The program returns a name match with the taxonomic status (accepted or not) and an overall matching score (a value between 0 (no match) and 1 (perfect match)). From this, two lists were produced: one identifying misspelled names and one identifying potential synonyms. The first list was generated by filtering out names with a taxonomic status as ”accepted”, an overall score below 1 and no partial match (i.e. both genus and species names are matched). The second list was created by filtering out (i) names with an overall score of 1 and whose submitted name was different from the accepted name, and (ii) names with an overall score under 1 and whose matched name was different from the accepted name. For both lists, we further screened the database names manually for the presence of the matched name (for the list of misspelled names) and for the presence of the accepted name (for the list of synonyms). The remaining and/or unresolved names were then scrutinized against the African Plant Checklist and Database (Klopper et al. 2007, African Plants Database 2015) and the World Checklist of Selected Plant Families (Govaerts et al. 2009) to assess their status.

Overall, if we consider records that passed the different georeferencing quality checks (see above), 3,114 species names (3806 taxa) were excluded from our database after these different standardization procedures.

Family names for angiosperms were standardized to following the Angiosperm Phylogeny Group III system (APG III 2009).

### Workflow to identify and merge record duplicates

The database is a compilation of both extensive ‘public’ databases compiled by herbarium institutes and smaller personal databases focusing on either a given taxonomic group or a given geographic area. Despite their limited number of records, the latter have been compiled by experts and therefore the quality of georeferencing and identification are generally better. A major issue was that most records in personal databases were duplicated within large herbarium database. Likewise, there was overlap in specimen data among major herbarium databases because specimens have often been collected in several duplicates that were later distributed among herbaria. It was important to identify and merge these duplicates because each could carry a different identification and/or georeference. Hence, the identification of duplicate records had to be carried out in order to select the most accurate information in cases where duplicate records contained conflicting data.

When duplicates with different identifications were encountered, the following procedure was followed to identify the most reliable record:

- if the identification varied between an institutional and a personal database, we chose the identification recorded in the personal database (see the description of the datasets below).
- if a personal database was not available, we chose the identification with the most recent date of identification.
- if identification dates were similar or not given, we chose the identification at the lowest taxonomic rank (e.g. genus, species, subspecies, etc.). For example, if one record was identified to the infra-specific level while another was identified to the genus level, then the former was chosen.
- if after these steps no one record was identified, a random one was chosen.

When duplicates with different coordinates were identified, several subsequent steps were undertaken to identify the most reliable georeferencing:

- if only one of the records passed the quality check for country described above, those coordinates were chosen.
- if the coordinates came from an institutional and a personal database, the chosen georeferencing was the one from a personal database (see the description of the datasets above).
- if none was chosen by the previous step, the chosen georeferencing was the one with the highest precision of the geographical coordinates using a precision code calculated for the project from 1 to 8 (see Table 1).
- if after these steps no one record was identified, a random one was chosen.

**Table 1.** Accuracy code given to georeferenced records and corresponding uncertainty in degrees.

Criterion	Code
accurate to degree only (~110 km)	1
15 minutes precision (~30 km)	2
5 minutes precision (~10 km)	3
2 minutes precision (~4 km)	4
minute is exact (~ 2 km)	5
1/10 decimal minute exact (~ 200 m)	6
1/100 decimal minute exact (~ 20 m)	7
1/1000 decimal minute exact (~ 2 m)	8

### Identification of introduced and cultivated taxa

Because we want to work only with natural occurrences of indigenous species, we had to, as far as possible, identify and discard specimens collected from planted and/or cultivated individuals and those from introduced species.

The first step in this process was to screen the text in the locality field of the specimen records. We first built a preliminary list of locality descriptions by searching for a list of keywords (e.g. ‘Botanical garden’). Of this preliminary list of 898 locality descriptions we selected 653 that most likely correspond to *ex situ* living collections. All records collected (1,427) from these localities were then discarded.

In order to differentiate between native species and cultivated or other introduced taxa, the following procedure was adopted. We expected to find most cultivated or introduced taxa among those with few collections (these taxa are in fact rarely collected in the field). We therefore first extracted all species with fewer than eleven records. Then, GBIF occurrences were used to document the distribution outside of the area covered by the RAINBIO database: for each species, we verified whether occurrences were available on GBIF and if that was the case, we downloaded GBIF occurrences using the *rgbif* package (Chamberlain et al. 2016). Species appearing as mostly collected outside of the geographical coverage of the RAINBIO database were selected and manually checked to confirm they were truly introduced/cultivated, resulting in a list of 1,658 cultivated/introduced species. This list was further completed by using information provided by the African Plant Checklist and Database (Klopper et al. 2007) and the World Checklist of Selected Plant Families (Govaerts et al. 2009). The final list of cultivated/introduced species identified within the RAINBIO database comprised 1,635 species, which corresponds fairly well to the ca. 2100 naturalized non-African species in the whole of Africa as calculated by Kleunen et al. (2015). All records belonging to those taxa were discarded (but see http://rainbio.cesab.org/ for the list of those species).

### Geographic coverage

Records of the RAINBIO database are localized in continental Africa, excluding Madagascar and Indian Ocean islands, but including Gulf of Guinea islands, Cape Verde and Zanzibar archipelagos representing 51 different countries. All records fall within an area delimited between -34.8328 and 37.1094 degrees of latitude, and between -25.33 and 51.4 degrees of longitude.

The geographic coverage of the RAINBIO database i.e. where record density is significant, is a region broadly delimited by ecoregions (*sensu* Olson et al. 2001) south of the Sahel and north of Southern Africa. The most significant amount of data originates from the tropical forest regions (Fig. 1).

### Taxonomic coverage

The RAINBIO database comprises 25,356 species of vascular plants and 29,659 taxa (including subspecies and varieties), 3,158 genera and 273 families. The list of all taxa recorded in the RAINBIO database can be found in the Appendices.

**Figure 3.**
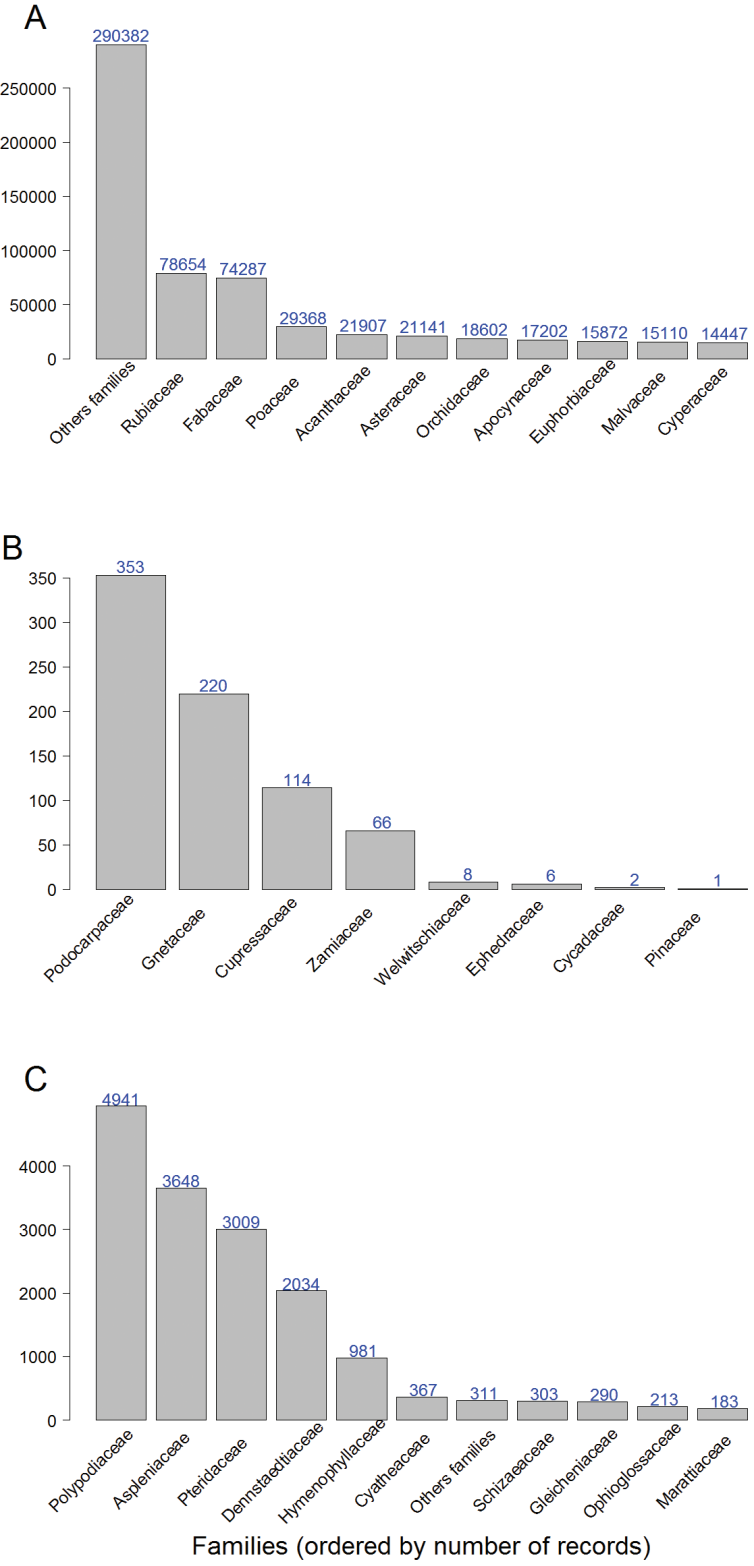
Most represented families (sorted by number of records) for each of the three divisions of vascular plants represented in the RAINBIO database. **A** Magnoliophyta **B** Gymnosperms **C** Pteridophyta.

Magnoliophyta are represented by 596,972 records and 24,770 species, Gymnosperms by 770 records and 40 species and Pteridophyta by 16,280 records and 546 species. The best represented families in Magnoliophyta are Rubiaceae, Fabaceae and Poaceae (Fig. 3A). Gymnosperms include eight families among which the Podocarpaceae is the most represented (Fig. 3B). Within Pteridophyta the most represented families are Polypodiaceae and Aspleniaceae (Fig. 3C).

In 2007, the total number of Angiosperm taxa in an area broadly corresponding to the geographic coverage of the RAINBIO database was estimated to be 32,424 by the African Plant Checklist and Database (Klopper et al. 2007, African Plants Database 2015). RAINBIO database comprises 29,013 angiosperm taxa. We can therefore estimate that the RAINBIO database includes information for approximately 89% of all known species in the area of interest.

### Habit data

We provide habit for almost all species recorded in the RAINBIO database (available for 23,111 species or 91% of all species). Information was gathered at the species level and was initially taken from the Naturalis Herbarium Collections database.

This information was then completed by relying on the field description of herbarium specimens: keywords for seven specific habits (tree, shrub, herb, liana, epiphyte, mycoheterotroph and parasitic) were searched for in the description field of all specimens. For example, for the ‘tree’ habit, the key-words were “Tree”,”tree”,”Arbre”,”arbre”,”Arbor”,”arbor”. If one of these key-words was found in the description field of a specimen, the record was tagged for the ‘tree habit’. The tags for each habit were then summed for each species. This procedure resulted for example in twenty tags for the species *Acacia adenocalyx* among which nine of them concerned the ‘shrub’ category and seven the ‘liana’ category. For each species the habit with the highest number of tags was chosen. If this habit represented less than half of the tags, the second ranked habit was considered as a secondary habit. For *Acacia adenocalyx*, this procedure therefore resulted in the choice of ‘shrub’ habit as the primary habit and ‘liana’ habit as a secondary habit. Erect palm-like plants (e.g. Palms, *Dracaena*, *Pandanus*) are included as ‘shrub’ or ‘tree’ according to literature.

The results obtained through this procedure were compared to the information obtained through the Naturalis Herbarium Collections database. Results were mostly congruent, validating our procedure. Mismatches between both sources and species with missing habit were finally manually checked and added by using information provided by the African Plant Checklist and Database (Klopper et al. 2007, African Plants Database 2015), the World Checklist of Selected Plant Families (Govaerts et al. 2009) and by checking specimens of such species.

### Temporal coverage

Collecting years range from 1782 to 2015.

### Description of the thirteen datasets

The thirteen datasets that contributed to the RAINBIO are described below and sorted according to the total number of record provided.

• **Naturalis Herbarium Collections database (WAG, L, U, AMD)**

Origin: Naturalis Biodiversity Center, Leiden, The Netherlands

References: Wieringa and Sosef (2011), Oever and Gofferjé (2012); http://herbarium.naturalis.nl

Metadata: http://vmamapgn-test.mpl.ird.fr/geonetwork/srv/eng/search#|8fa7bdc6-69a6-4ccd-a3c9-ea8a1836ac0e

Access date: first extract in 10/2014, followed by an update in 08/2015.

Type: herbarium specimens

Query: Africa, excluding some countries (Madagascar & north African countries)

Number of records included: 519,623

• **Missouri Botanical Garden database (MO)**

Origin: TROPICOS database, Missouri Botanical Garden, Saint Louis, U.S.A.

References: http://www.tropicos.org

Metadata: http://vmamapgn-test.mpl.ird.fr/geonetwork/srv/eng/search#|513617b3-9d01-4c0d-8909-f965ff3eed53

Access date: 7/11/2014

Type: herbarium specimens

Query: excluding several African countries (South Africa, Madagascar, North African countries). The tag ‘is_cultivated’ was False.

Number of records included: 147,520

• **Meise Botanic Garden database (BR)**

Origin: Botanic Garden Meise, Meise, Belgium

References:

http://www.br.fgov.be/RESEARCH/COLLECTIONS/HERBARIUM/advancedsearch.php

Metadata: http://vmamapgn-test.mpl.ird.fr/geonetwork/srv/eng/search#|b5e8416d-d742-4cef-99be-75e646cfb041

Access date: October 2014

Type: herbarium specimens

Number of records included: 132,771

• **Université Libre de Bruxelles herbarium database (BRLU)**

Origin: database of the Herbarium of the Université Libre de Bruxelles, Brussels, Belgium

References: http://herbarium.ulb.ac.be/

Metadata: http://vmamapgn-test.mpl.ird.fr/geonetwork/srv/eng/search#|87177d1b-54f4-4ca2-a7de-a5db91f8b605

Access date: November 2014

Type: herbarium specimens

Number of records included: 62,380

• **Royal Botanic Gardens, Kew (K)**

Origin: downloaded from gbif.org.

References: http://www.gbif.org/dataset/cd6e21c8-9e8a-493a-8a76-fbf7862069e5

Metadata: http://vmamapgn-test.mpl.ird.fr/geonetwork/srv/eng/search#|ac585ae5-b331-4855-873c-f78a92919f5c

Access date: 9/12/2014

Type: herbarium specimens

Query: only georeferenced records in African countries

Number of records included: 55,919

• **Collection of African plant samples dried in silica-gel**

Origin: Evolutionary Biology and Ecology Unit, Université Libre de Bruxelles, Brussels, Belgium.

Metadata: http://vmamapgn-test.mpl.ird.fr/geonetwork/srv/eng/search#|e43766e7-e734-487d-98f4-a45188d99edd

Access date: November 2014

Type: leaves dried in silica-gel (no voucher)

Number of records included: 14,510

• **Instituto de Investigação Científica Tropical (LISC), University of Lisbon**

Origin: downloaded from gbif.org

References and metadata: http://www.gbif.org/dataset/231c5bcf-1b56-4905-a398-6d0e18f6de1a

Access date: 24/7/2015

Type: herbarium specimens

Query: only georeferenced records in African countries

Number of records included: 14,301

• **Occurrences from tree plot data**

Origin: Evolutionary Biology and Ecology Unit, Université Libre de Bruxelles, Brussels, Belgium

References: (Dauby 2012; Dauby et al. 2014)

Metadata: http://vmamapgn-test.mpl.ird.fr/geonetwork/srv/eng/search#|a7e8a9d5-1cfe-4ee1-a16a-effa81dd34bf

Access date: November 2014

Type: Inventory tree data.

Number of records included: 12,874

• **African Palms**

Origin: database collated by Anne Blach-Overgaard

References: (Blach-Overgaard et al. 2010; Blach-Overgaard et al. 2013; Blach-Overgaard et al. 2015)

Metadata: http://vmamapgn-test.mpl.ird.fr/geonetwork/srv/eng/search#|f20ff0ad-94a5-450f-9ea2-28af706a1b40

Access date: November 2014

Type: herbarium specimens

Number of records included: 5,567

• **Rubiaceae endemic to Atlantic Central Africa**

Origin: database collated by Bonaventure Sonké and Vincent Droissart

References: (Droissart et al. 2011)

Metadata: http://vmamapgn-test.mpl.ird.fr/geonetwork/srv/eng/search#|1bd345aa-a9eb-4be9-8cc4-1c5869118105

Access date: November 2014

Type: herbarium specimens

Number of records included: 4,529

• **Dzanga-Sangha vascular plant database**

Origin: database collated by David J. Harris in the Dzanga-Sangha region (Central African Republic)

References: (Harris 2002)

Metadata: http://vmamapgn-test.mpl.ird.fr/geonetwork/srv/eng/search#|07425bb4-f412-4961-9ac0-34c3445027f9

Access date: November 2014

Type: herbarium specimens

Number of records included: 3,571

• **Orchidaceae endemic to Atlantic Central Africa**

Origin: database collated by Vincent Droissart and Tariq Stévart

References: (Droissart 2009; Droissart et al. 2011)

Metadata: http://vmamapgn-test.mpl.ird.fr/geonetwork/srv/eng/search#|1aea0fd6-f074-4274-878c-38d755a278b4

Access date: November 2014

Type: herbarium specimens

Number of records included: 2,054

• **African *Berlinia* (Caesalpinioideae)**

Origin: database collated by Barbara Mackinder

References: (Mackinder and Harris 2006, Mackinder and Pennington 2011)

Metadata: http://vmamapgn-test.mpl.ird.fr/geonetwork/srv/eng/search#|05579478-47d2-421e-94fa-b1476a39a133

Access date: November 2014

Type: herbarium specimens

Number of records included: 1,596
